# Mood and the Market: Can Press Reports of Investors' Mood Predict Stock Prices?

**DOI:** 10.1371/journal.pone.0072031

**Published:** 2013-08-28

**Authors:** Yochi Cohen-Charash, Charles A. Scherbaum, John D. Kammeyer-Mueller, Barry M. Staw

**Affiliations:** 1 Department of Psychology, Baruch College and Graduate Center, City University of New York, New York, New York, United States of America; 2 Warrington College of Business Administration, University of Florida, Gainesville, Gainesville, Florida, United States of America; 3 Haas School of Business, University of California, Berkeley, California, United States of America; Universidad Veracruzana, Mexico

## Abstract

We examined whether press reports on the collective mood of investors can predict changes in stock prices. We collected data on the use of emotion words in newspaper reports on traders' affect, coded these emotion words according to their location on an affective circumplex in terms of pleasantness and activation level, and created indices of collective mood for each trading day. Then, by using time series analyses, we examined whether these mood indices, depicting investors' emotion on a given trading day, could predict the next day's opening price of the stock market. The strongest findings showed that activated pleasant mood predicted increases in NASDAQ prices, while activated unpleasant mood predicted decreases in NASDAQ prices. We conclude that both valence and activation levels of collective mood are important in predicting trend continuation in stock prices.

## Introduction

“Once Again, Fear Sends Stocks Down” (New York Times, August, 20, 2011)

“In the absence of major economic news, stock rode a tail wind of optimism …” New York Times, August 6, 2012)

U.S. Markets Fidget, Fret, and Go Nowhere (New York Times, November 10, 2012)

“The market is extremely skittish right now, that's why we're seeing such big movers.” (New York Times, February 5, 2013)

Daily news reports about the stock market commonly refer to more than changes in the economy or the announcement of corporate earnings. They often describe the “mood” of the market or stock traders in emotional terms such as “anxious,” “depressed,” “calm” or “enthusiastic.” These affective descriptors go beyond the dueling forces of “fear” and “greed” that have long been used by pundits and stock analysts to depict market psychology (e.g., [1], [2]). They are also more ubiquitous than some famous descriptions of financial markets in emotional terms, such as when Federal Reserve Chairman Alan Greenspan once proclaimed that the stock market was exhibiting “irrational exuberance” [3].

Despite the omnipresence of mood in descriptions of traders in the stock market, scientific study of the relationship between the collective mood of traders and stock-market performance has been lacking. To fill this gap, we report on a study examining the relationship of investors' mood to stock market behavior. Of primary focus will be the question of whether traders' collective mood, as reported in newspapers, can actually predict future increases or decreases in stock prices.

## The Collective Mood of Investors

Affective states such as mood and emotion are no longer considered to be solely an individual-level experience, but also a group (e.g., [4], [5]) and collective experience [6], [7]. For example, affective states have been shown to influence decision-making of individuals (e.g., [8] and groups [9]. As a result, our major assumption is that, through their influence on trading decisions and behavior of large groups of traders, press reports on collective mood may influence the stock market's behavior. In this study we will specifically examine the relationship of reported collective mood to stock market behavior, and particularly whether such reports of affective states can predict market movements.

### Investor mood vs. investor sentiment and other mood proxies

Moods are diffused affective states that can originate from external events, prior emotional experiences, or the internal disposition of the person [10]. Although moods are mostly global (that is, characterized as positive or negative, happy or sad), they can also be more specific, such as an angry or joyful mood [10]. As internal states of the people experiencing them, moods are typically assessed using self-report measures asking respondents about their affective states [11], by taking physiological measurements (for a review see [12]), or by the observations of others' facial expressions and behaviors (e.g., [13], [14]). Mood is commonly represented by various circumplex models (e.g., [15], [16], [17]), and in this study we rely on the model suggested by Larsen & Diener [18]. Their affective circumplex is composed of two dimensions: pleasantness and activation. Together, these two dimensions form four quadrants of mood: high activation and pleasantness (e.g., enthusiasm), low activation and pleasantness (e.g., calmness), low activation and unpleasantness (e.g., depression), and high activation and unpleasantness (e.g., anxiety).

In contrast to mood, investor sentiment is generally defined as “a belief about future cash flows and investment risks that is not justified by the facts at hand” ([19], p. 129). For example, Fisher and Statman [20] describe the sentiment of large investors based on Merrill Lynch's definition as “the mean allocation to stock in their recommended portfolios” ([9], p. 16). Another indicator of sentiment comes from the publication, Investor Intelligence, using a survey of newsletter writers and their weekly classification as *Bullish*, *Bearish*, and *Waiting for Corrections*. Similarly, the sentiment of small investors has been assessed using sources such as the American Association of Individual Investors (AAII), which conducts weekly sentiment surveys among its members, asking them to classify themselves as *Bullish*, *Bearish*, and *Neutral*. Finally, there are objective indicators of sentiment such as the ratio of put to call options and measures of market volatility, such as the Chicago Board Options Exchange Market Volatility Index (or VIX), commonly called the “fear index.”

Unfortunately, terms such “sentiment” and “fear index” may be misleading because they are not based on actual measures of affect (e.g., fear). Also, these measures are often circular because they commonly comprise previous investment behavior (e.g., allocation to common stocks or purchase of stock options) rather than non-market (or emotional) indicators. And, when they are based on perceptions of future market performance (e.g., bullishness surveys), they are difficult to separate from real changes in economic and market conditions [19]. Thus, it is not clear whether sentiment indicators are causes or effects of the market – or whether as Nofsinger [6] claims, the market is an indicator of social mood, and social mood directly influences the market.

In addition to investor sentiments, researchers have identified some rather indirect measures of mood or “mood proxies” [21] and examined their ability to predict financial markets. For example, results of sporting events [22], seasonality (SAD; e.g., [23]) and the weather (for a review see [21]) are mood proxies that have been investigated as predictors of market behavior. However, because the relationship between these factors and actual moods is relatively weak (e.g., [24], [25]–[27]), it is doubtful whether any of them are strong indicators of investor mood. By contrast, for this study we developed a theoretically based measure of collective mood that will allow us to examine how reported collective mood might actually predict market movements.

## Mass Media, Collective Mood, and the Stock Market

Collective (or social) mood is the aggregate mood of individuals [7]. We view collective mood as a population-level variable, in that it represents the mood of large groupings, such as nations, professions, and the pool of active investors in the stock market. Similar to group emotion [5], collective mood can be created by processes such as contagion, vicarious affect, and shared affective experiences. For example, world events, changes in business conditions, and even widespread fashions or fads can be simultaneously experienced by investor groups and lead to common affective reactions [28]. Alternatively, moods can be experienced by a particular subgroup and then spread to others through direct social interaction, social networks, or the media. In this study we will emphasize the spread of information about mood through the mass media.

Although much research has documented the spread of affective reactions via unconscious mimicry of others' facial expressions, vocal tone, and body postures within a shared physical space [29], [30], mood can also spread among individuals who are not in direct contact. One major tool for spreading information, and hence creating collective mood, is the mass media. For example, research has found that watching the news can impair or repair peoples' moods [31], [32]; that newspaper headlines regarding national events, such as war, are significantly related to population depression [33]; and that reading a sad or a happy story in one domain can affect overall mood, while reading about specific risks can increase overall risk perception [34]. Thus, the influence of news can spill over across domains, influencing the general mood of the population as well as moods pertaining to a specific domain like the stock market.

There have been some previous studies of the effect of mass media on the stock market. For example, Nofsinger [35] found that press releases about particular stocks increased the trading volume of those stocks, and that the release of macro-level economic news increased the volume of trading in general. Researchers also found that commentators' use of active or passive metaphors when describing the market's activity (e.g., the Dow fell vs. the Dow was pushed down) and the way trends were presented (i.e., in a tabular vs. a graphic form), influenced investors' perception of market trends [36]. Unfortunately, media exposure may not always benefit investors. For example, reading news reports about the market and specific stocks was found to lead to more financial losses than not reading such reports [37]. The problem may be that financial news can falsely lead investors to believe in trend continuance. Investors may erroneously believe that market and stock news reflect stable and causal reasons for a loss or a gain in the market or in a specific stock [38], leading them to buy when prices are high and to sell when prices are low.

One recent study examined the linkage between the negativity of media reporting and subsequent changes in the stock market. Tetlock [39] identified a Pessimism Factor in the reports of the Wall Street Journal's *Abreast of the Market* column collected over a 16 year period. The Pessimism Factor was primarily composed of words associated with a negative outlook, words implying weakness, words associated with failure, and words associated with falling movement. Tetlock found that this Pessimism Factor was (a) predicted by low Dow Jones returns, (b) predicted next-day downward movement in the Dow Jones, which was reversed 2–5 days later, and (c) at very high or very low levels, predicted high trading volume in the Dow Jones. Tetlock further found that the Pessimism Factor had a more profound and a longer lasting influence on small stocks, as compared to stocks included in the Dow Jones Industrial Average.

Although Tetlock [39] viewed his Pessimism Factor as an indicator of market sentiment, this measure may be as much a content-based indicator as one that is affectively based. It relies primarily on descriptions and interpretations of prior market movements rather than a simple aggregation of affective reactions to these events. Nonetheless, Tetlock's study demonstrated that media interpretation of market events (via an influential newspaper column) can affect subsequent market movements. As a result, we might expect an affect-focused measure of moods to be more valid for predicting market behavior from investors' collective mood.

Based on the above literature, we believe that the collective mood of investors is influenced, at least in part, by news reports. Such reports not only provide descriptions of previous market behavior, but also an affective interpretation of the trading day. Both the news and the affective tone of these reports may be shared directly with readers and indirectly via interactions between readers and others in their social environment. Ultimately, such a diffusion of both information and affect may lead the larger population of investors to react to the market in similar ways. It may also contribute to the inter-correlation of individual stocks and stock indices, as investors tend to view the financial landscape with a similar perspective or bias [40], [41]. The end product of such diffusion can be a generalized tendency to increase or decrease risk (or a common desire to enter or leave the market), often referred to as “herd behavior” (e.g., [42]).

## Mood and Trading Behavior

We next examine specific issues of the relationship between mood, decision-making, and trading behavior as they relate to our particular research questions: (a) can press reports on collective mood predict market behavior? (b) what is the relationship between reported mood pleasantness and market outcomes? and (c) what is the relationship between reported mood activation-level and market outcomes?

### Can press reports on traders' collective mood predict subsequent market behavior?

Past research has found that mood influences the trading behavior of individual investors [43]–[46]. Theoretical explanations for the influence of mood on investors' trading behavior emphasize the influence of moods and emotions on decision-making and risk-taking. Such influences can occur by anticipatory emotions, such as fear and hope experienced at the time of decision-making [47], [48], by decision-related stress [49], and by anticipated emotions, such as regret, which investors want to avoid (e.g., [50]). Extrapolating from the influence of mood on trading behavior of individuals, we therefore postulate that collective mood predicts stock-market performance. Through its influence on the behavior of multiple traders, collective mood may lead to changes in market behavior.

### What is the relationship between reported mood and market performance?

To answer this question, we focus on the relationship between mood and decision-making. Despite a diversity of theories and findings on the topic, it is widely agreed that mood influences decision-making directly and indirectly through its effects on cognitive processes such as perception, forecasting, goal-setting, and motivation (for reviews see [8], [51], [52], [53]). Some of the major theoretical models supporting a relationship between mood and decision-making are the mood as information model, the associative network theory, and the evolutionary theory of hedonism.

According to the mood as information model (e.g., [54], [55]), moods provide congruent information to decision-makers, such that pleasant moods imply that one's world is safe, and unpleasant moods imply that one's world is in danger. This informational function of moods is exacerbated by congruent attention and recall processes, emphasized by the associative network theory [56]. Thus, when in a pleasant mood people have more positive memories, they perceive neutral stimuli as more positive, and their attention is directed at more positive stimuli. When in an unpleasant mood peoples' memories are more negative, neutral stimuli are perceived as more negative, and attention is directed at more negative stimuli. Pleasant and unpleasant moods also have different implications for motivation. Whereas most people in pleasant moods will strive to maintain these states [57]–[59], people in unpleasant moods will strive to repair their mood state (e.g., [60]). And, in accordance with the evolutionary perspective, people are motivated toward approaching situations that benefit them and avoiding situations that might harm them in the future [61].

Based on these underlying theoretical perspectives, it is possible to offer competing hypotheses regarding the relationship between mood pleasantness and individual trading behavior, and by extension, between collective mood pleasantness and market performance. Specifically, it is possible to predict both a positive and a negative relationship between mood pleasantness and stock market behavior.

### Positive relationship between mood pleasantness and market performance

A large body of research has shown mood congruency in attention, perception, and recall (e.g., [56], [62], [63]), such that people in a pleasant mood perceive situations as more positive, and in an unpleasant mood see the situation as more negative. This informational function of mood influences two major factors that relate to trading decisions: decision-making strategies and risk perception.

#### Mood and decision-making strategy

It has been hypothesized that because pleasant mood signals safety and unpleasant mood signals danger, decision-makers are less vigilant and use more heuristics in their decision-making processes when in a pleasant mood, as compared to when decision makers are in an unpleasant mood [64]. For example, people are more likely to use the availability heuristic [65] when in a pleasant mood, evaluating future events based on the salience of information to them at the time. Thus, De Bondt [66] found that non-professional investors tend to rely on past performance to predict future performance, such that when past market performance is bullish or bearish, they predict future market performance to follow suit. The fact that the influence of mood is considered to be strongest when the mood is related to a relevant event (for a review see [67]), means that mood derived from previous market behavior will have more congruent influence on future behavior than incidental mood, unrelated to market behavior.

#### Mood, risk perception, and risk taking

Researchers have also found mood congruency in people's predictions about the future. When they are in a pleasant mood people are optimistic, and when in an unpleasant mood they are more pessimistic about the future [34], [68]. Similarly, in a pleasant mood people perceive risk to be low and in an unpleasant mood they perceive risk to be high [69], [70]. Moreover, it has also been found that when decision-makers are in a pleasant mood they perceive events as opportunities, and when they are in an unpleasant mood they perceive events as threats [71]. These findings imply that, as mood is reported to be pleasant, the market is perceived to be less dangerous, and investors are more likely to commit funds to the market, driving stock prices higher. By the same token, so as to avoid any further risk in situations perceived as threats [72], investors learning about unpleasant mood in the market, will be inclined to sell their assets or buy only at lower prices, thus leading to market declines.

Not only can we predict that mood influences the perception of risk, but there are data indicating that mood can also affect risk taking behavior (e.g., [73]), especially when people perceive the level of risk level to be low (e.g., [74], [75]). In the investment context, Au et al. [43] found that investors in a pleasant mood were overconfident and took higher risks. Much less research has been devoted to unpleasant mood, but recently it has been found that people in a depressed mood are less willing to take risks, as compared with those in neutral or pleasant moods [76], and this risk aversion can result in lower returns [77].

From prior research and theory we can expect that investors in a pleasant mood will perceive the stock-market as a relatively safe place and one that can promote future positive outcomes, thereby increasing the likelihood that they will stay in the market and/or increase their investments in it. By the same principle, investors in an unpleasant mood will be more likely to perceive the market as a dangerous place, one that can potentially harm their well-being, thereby making it more likely that they will sell stocks and/or reduce their involvement in the market. Therefore, we hypothesize that reports of investors' mood on Day 1 predict trend continuance in market performance on Day 2. That is, reported pleasant mood on Day 1 may predict market gains at the opening of the following trading day, and reported unpleasant mood on Day 1 may predict market losses at the opening of the following trading day (Hypothesis 1a).

### Negative relationship between mood pleasantness and market performance

Mood management models and research findings can also lead to the hypothesis that there may be a negative relationship between mood and trading behavior. Some studies show mood incongruent processes that serve to maintain or repair one's mood. For example, in naturalistic settings (as opposed to lab settings), and when they are not aware of their mood, people show mood incongruent recall [78]. According to Forgas [79], [80], mood incongruent responding is a spontaneous mood management strategy designed to achieve affect control. Thus, whereas initial responding is mood congruent, subsequent responses can be mood incongruent. If mood incongruent cognitive processes occur in naturalistic settings where people do not expect their mood to be related to their decisions, it is possible that mood incongruent effects will occur in the context of trading decisions.

Mood incongruent processes imply a negative relationship between mood and market performance. Similar to our previous discussion, we divide the arguments about the negative relationship between mood pleasantness and stock market behavior into effects of decision-making strategies and risk taking behavior.

#### Mood and decision-making strategy

Although many studies have found that those in a pleasant mood are prone to making mistakes and are more susceptible to cognitive biases (e.g., [81], [82]), other research has shown that those in a pleasant mood are more vigilant and effective decision-makers (e.g., [51], [83], [84]–[88]). Since savvy trading involves buying low and selling high, one might therefore predict that a reported pleasant mood can lead traders to sell stocks when prices are high, resulting in a negative relationship between reported mood and subsequent market behavior.

#### Mood, risk perception, and risk taking

In contrast to findings (cited earlier) showing pleasant mood to lead to lower risk perception and unpleasant mood leading to higher risk perception, other research has demonstrated that a pleasant mood leads people to focus more on the harming aspects of potential loss than on the benefits of gain, thus increasing risk aversion. This research is based on the mood maintenance model, according to which people want to preserve their pleasant states [89]. Such risk aversion among those in a pleasant mood occurs especially when stakes are high and investors have a lot to lose. This research also shows more risk proneness among happy people, but only when the stakes are low and people have less to lose and potentially something to gain [74], [75], [90]–[92]. Thus, in the context of the stock market, positive mood coupled with a high risk of future loss, might actually lead to risk-averse behavior, and subsequent withdrawal from the market. Indeed, Liao, Huang, and Wu [93] recently found that when investor optimism (as measured using a combination of traditional investor sentiment indicators) is high, fund managers tend to sell, thus counteracting the sentiment and leading to market declines.

As for decision-makers who experience unpleasant moods, it has been shown that distressed decision-makers are willing to take higher risks than non-distressed decision-makers, and are willing to gamble more money when they perceive good chances to win (and hence repair their negative mood, [94]). Similar results were obtained with respect to risky strategic decisions. When they perceived higher chances to make a profit, decision-makers in an unpleasant mood were more willing to take risks than decision-makers in a pleasant mood [71], [95]. Porcelli and Delgado [96] also showed that stress leads to higher risk-taking in gambling and more reliance on intuitive rather than systematic decision-making. Therefore, in terms of stock trading, reported unpleasant moods may lead to an increased tendency to take risks in the market, driving prices higher. Unpleasant mood created by a down market may set in motion processes that result in a future rise in stock prices.

Cumulatively, these mood management and risk taking models may be used to predict trend reversals in the stock market. That is pleasant traders' mood might portend a market decline, and when the general mood is unpleasant, there could be greater likelihood of a market increase. Thus, we can hypothesize that reports of investor mood at Day 1 predict trend reversal in market performance on Day 2. That is, reported pleasant mood on Day 1 may predict a market decline at the beginning of the following trading day, and reported unpleasant mood on Day 1 may predict a market increase at the beginning of the following trading Day (Hypothesis 1b).

### What is the relationship between mood activation and market performance?

Whereas most studies examining mood and decision-making focus on its valence (or degree of pleasantness), there is good reason to believe that a mood's activation level (or arousal) may also influence decision-making. For example, M. S. Clark [97] claimed that arousal, similar to valence, is another unit of information stored in memory and retrieved when an event occurs [56], [62]. As such, the greater the arousal one experiences in certain mood states, the more intense is the priming effect of this mood on recall, perception, and behavior. In other words, high levels of arousal impact behavior more than low levels of arousal and intensify the influence of mood on behavior.

Arousal levels have been hypothesized to be responsible for various phenomena, such as panic attacks [98], reduced confidence levels [99], as well as reduced performance. Mann [49] provided examples of high arousal levels leading to poor decision-making, regardless of the valence of moods or emotions. Arousal has also been found to impact risk-taking. Researchers found that anger, which is high in arousal, led to greater risk-taking [100], [101]. Mano [72], [94] also found that arousal level, more so than mood valence, led to higher risk-taking. According to Mano, this is because higher arousal narrows attention, leading to simplified decision-making processes and to more extreme judgments. Nonetheless, the relationship between arousal and risk taking may not be so consistent. Mano [72] showed that when the stakes were high, the combination of high arousal and negative valence (distress), led participants to be risk-averse, but participants experiencing calmness (low arousal and positive valence) were more risk-seeking. Similar findings have been demonstrated regarding acute physiological stress [96]: participants under stress took less risky decisions when chances to gain were greater than were chances to lose and more risky decisions when chances to lose were higher than were chances to gain.

In the investment context, Lo, Repin, and Steenbarger [45] followed a sample of day-traders who invested their own money and completed mood surveys at the end of each trading day. These researchers found that mood arousal level was uncorrelated with trading performance, but that higher levels of pleasant or unpleasant mood were related to bad trading performance. Lo et al. explained these results by citing the disrupting influence of affect on complex decision-making. Similarly, Shiv, Loewenstein, Bechara, Damasio, and Damasio [102] compared normal to brain damaged participants in a gambling task. They showed that those participants with damage in brain areas responsible for emotional functioning performed better, regardless of previous wins or losses, as compared to normal and control participants. According to these results, affect (and particularly arousal) may interfere with making sound investment decisions.

Given the conflicting findings on arousal, we can only make rather speculative two-way predictions regarding the relationship between activation level and trading behavior. Given prior theory and data, we can predict that mood activation at Day 1 might predict either an increase or decrease in risk taking and hence stock prices (Hypotheses 2a, 2b). This is because activation may play a facilitating role (along with mood valence) in either trend continuance or reversal. In trend continuance, a positive market that engenders strongly activated positive moods on Day 1 would likely lead to continued market gains at the opening of the following trading day. Likewise, a negative market that engenders strongly activated unpleasant moods on Day 1 might lead to continued market losses at the opening of the following trading day. With trend reversal, the level of mood activation might heighten a negative relationship between mood at Day 1 and next day's prices. Unlike the hypotheses for the pleasantness dimension of affect, however, our predictions involving mood activation should be considered more exploratory than confirmative

## Methods

The reported mood data in this study consisted of emotion-laden words from newspaper reports of the stock market and investor behavior. Market behavior data consisted of opening and closing stock prices, as measured by standard market indices such as the NASDAQ Composite Index (NASDAQ), the Standard and Poor's 500 Index (S&P 500), and the Dow Jones Industrial Average (Dow). Data collection was conducted in four stages. First, we created a list of emotion words from daily reports of positive and negative market action. Then, with the help of independent raters, we placed these emotion words on an affective circumplex, forming mood indices for use in subsequent analyses. Third, we collected a sample of emotion words used in newspaper reports on the stock market for each day of a calendar year. Fourth, we collected the daily opening and closing prices of various stock market indices for the same year. These mood and stock price data were then analyzed using time-series analyses. We describe these stages of the research in more detail below.

### Development of the emotion word list

Since most lists of emotion words are designed to assess emotion in a broad range of situations (e.g., from personal achievement to interpersonal relationships), we constructed a list of emotion words that would likely be more relevant to financial contexts and, in particular, investing in the stock market. Therefore, to create a list of emotion words for our data collection we (a) examined a sample of trading days from which to collect these words, and (b) selected the emotion words from the newspaper reports of the market performance on the days sampled.

#### Selection of trading days

For the creation of the initial “emotion word bank,” we chose trading days between October 1987 and January 2001 that were positive, negative, or neutral in terms of market performance. We defined the nature of the trading day by the movement (up or down) in both the Dow and the NASDAQ on a given day. We chose the days with highest gains and steepest falls in the Dow using the Greatest Net Gain/Loss measures published on the Dow Jones web site. We then examined the performance of the NASDAQ on the same days. If the change in the NASDAQ was greater than 1% and in the same direction as the Dow, we included that day in our pilot sample. Overall, our sample included 10 days of steep market increases (ranging from 1.8% to 5.0% in the Dow, and from 1.9% to 14.2% in the NASDAQ) and 10 days of steep market decreases (ranging from −2.8% to −22.61% in the Dow and from −1.2% to −11.35% in the NASDAQ). Neutral trading days were defined as those with a market change of less than 1% in both the Dow and the NASDAQ. We used a random number table to select 10 neutral trading days from this pool, yielding a sample with market changes ranging from 0.1% to 0.6% in the Dow and from 0% to 0.9% in the NASDAQ.

#### Selection of emotion words

Using the Lexis-Nexis database, we searched newspaper articles describing the stock market during the 30 days in our pilot sample. We examined articles from the five highest circulating newspapers in the United States: Wall Street Journal (WSJ), USA Today, New York Times, Los Angeles Times, and Washington Post. Newspaper circulation was determined by using the 2000 Editor & Publisher Year Book.

Using Lexis-Nexis Academic and a manual search, we examined articles in these newspapers that were published on the day following each of the 30 trading days in our pilot sample. We searched for articles that contained the keywords of Dow or NASDAQ in the headline or the lead paragraphs (We searched the WSJ manually, because articles from the WSJ did not appear in full in the Lexis-Nexis database). The number of relevant articles ranged from 2 to 20 per day with an average of seven articles a day. We read each selected article and identified all the words in it that described an emotional state of traders or trading activity. Only words that described the previous day's trading activity and traders' reactions were recorded. This search produced a list of more than 200 unique emotion words. To condense the list we retained only words that appeared two or more times in at least one group of trading dates (i.e., highly positive, highly negative, and neutral). The final list consisted of 80 unique emotion words.

### Placing the words in the affective circumplex

To create theoretically meaningful mood indices to be used for data analysis, we clustered the emotion words based on the affective circumplex [18]. For this purpose, 21 independent raters used the affect grid [103] to rate each of the 80 words in our list, coding for its location on the grid. The raters were volunteers; 16 were emotion researchers recruited through an ad in the Emonet listserv (an electronic list serving researchers of emotions in the organizational context). These are considered Subject Matter Experts (SME). Additional raters were three graduate students and two non-academic individuals.

**Figure 1:**
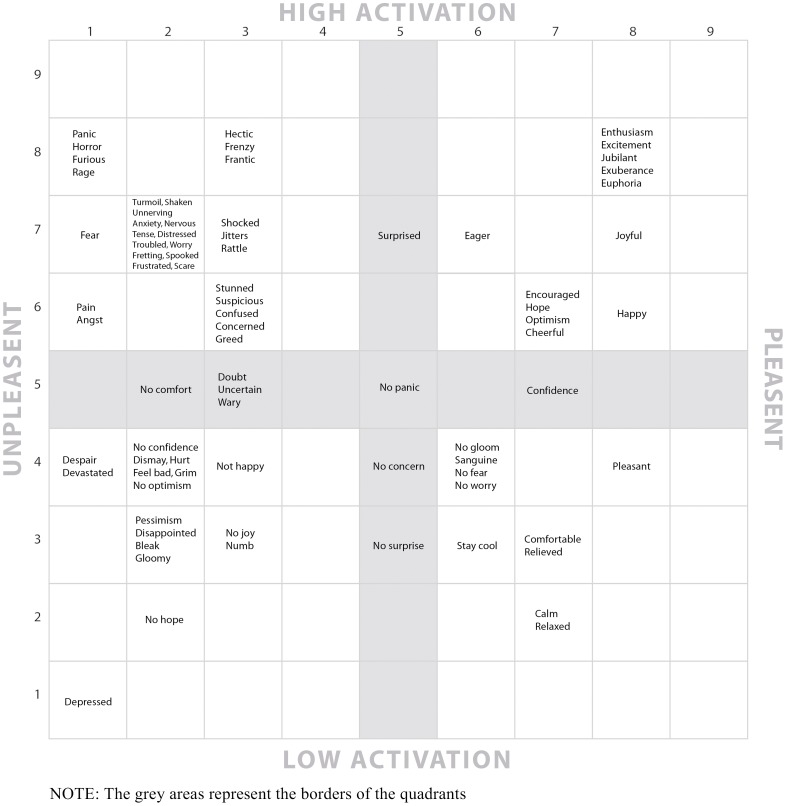
An affect circumplex based map of emotional states in the stock-market.

We computed interrater agreement for pleasantness and activation scores separately. Interrater agreements for both the activation and the pleasantness scores were high (average measure ICC = 0.99; 0.98 for pleasantness and activation respectively). We also compared the ICCs for the SME and non-SME respondents, for pleasantness and activation separately. ICCs were high in all cases (for pleasantness, ICC = 0.99, 0.97 for SME and non-SME respectively; for activation, ICC = 0.97, 0.90 for SME and for non-SME respectively.) For each emotion rating, we also examined mean differences of ratings between the SME and non-SME raters. Except for two exceptions in the activation dimension and three exceptions in the pleasantness dimension, there were no significant differences between the ratings of the SME and non-SME respondents. Significant differences were found for the activation dimension of ‘cheerful’ (mean = 7.12, 6.2 for SME and non-SME respectively) and of ‘rage’ (mean = 8.75, 9.00 for SME and non-SME respectively). As for the pleasantness dimension, significant differences were found for ‘no-joy’ (mean = 3.47, 1.60 for SME and non-SME respectively), for angst (mean = 1.57, 2.40 for SME and non-SME respectively), and for “optimism” (mean = 7.60, 8.60 for SME and non-SME respectively). Given the high levels of ICC and the low number of mean differences between SME and non-SME raters, we concluded there were no significant differences in the ratings of SME and non-SME respondents, and therefore combined the ratings of both samples. We used the average scores of each emotion word on each dimension to place the words in their appropriate location in the affect grid [103], essentially creating a map of emotional states in the stock-market context (see Figure 1). As can be seen in Figure 1, the distribution of emotion words across the 81 cells of the grid is not even, some cells having more words in them than others.

### Mood indices

Placing the emotion words in the affective circumplex allowed us to create meaningful mood indices to be used for our data analysis. To create these indices, we combined the 80 emotion words according to their location on the affect grid. For example, we combined all the emotions that are part of the low activation - pleasant affect quadrant of the grid to create an index of deactivated pleasant emotions. Because the number of emotion words varied across quadrants, we used the mean as our method of combining these data. For example, if a quadrant had 10 emotion words in it we summed the number of appearances of each word and divided it by the total number of words (i.e., 10). Overall, we created the following eight reported mood indices:

#### Activated unpleasant mood index

This index was composed of 31 items that are located in the corresponding quadrant in the affect grid (angst, anxiety, cautious, concerned, confused, distressed, fear, frantic, frenzy, fretting, frustrated, furious, greed, hectic, horror, jitters, nervous, pain, panic, rage, rattle, scare, shaken, shocked, spooked, stunned, suspicious, tense, troubled, turmoil, and worry; please see Figure 1). This index did not include the unclassified items in the ‘borders’ of the quadrant (i.e., no comfort, doubt, uncertain, wary, surprise, and no panic), since these words could have been placed on more than one index.

#### Activated pleasant mood index

This index was composed of 13 items that are located in the corresponding quadrant in the affect grid (cheerful, confidence, eager, encouraged, enthusiasm, euphoria, excitement, exuberance, happy, hope, joyful, jubilant, and optimism; please see Figure 1). This index did not include the unclassified items in its quadrant's borders (i.e., confidence, surprise, and no panic), as these could have been placed on more than one index.

#### Deactivated unpleasant mood index

This index was composed of 17 items that are located in the corresponding quadrant in the affect grid (bleak, depressed, despair, devastated, disappointed, dismay, feel bad, gloomy, grim, hurt, no confidence, no hope, no joy, no optimism, not happy, numb, and pessimism; please see Figure 1). This index did not include the unclassified items in its quadrant's ‘borders’ (i.e., no comfort, doubt, uncertain, wary, no concern, and no surprise), as these could have been placed on more than one index.

#### Deactivated pleasant mood index

This index was composed of 10 items that are located in the corresponding quadrant in the affect grid (calm, comfortable, no fear, no gloom, no worry, pleasant, relaxed, relieved, sanguine, and stay cool; please see Figure 1). This index did not include the unclassified items in its quadrant's ‘borders’ (i.e., confidence, no concern, and no surprise), since these could have been placed on more than one quadrant.

#### Pleasant mood index

This index included all the items representing pleasantness, regardless of their activation level. That is, it included all the items in the Activated Pleasant quadrant and the items in the Deactivated Pleasant quadrant, as well as the unclassified, ‘border’ item “confidence.” Altogether, this index was composed of 23 items.

#### Unpleasant mood index

This index included all the items representing unpleasantness, regardless of their activation level. That is, it included all the items in the Activated Unpleasant quadrant and the items in the Deactivated Unpleasant quadrant, as well as the unclassified, ‘border’ items: no comfort, doubt, uncertain, and wary. Altogether, this index was composed of 53 items.

#### High activation mood index

This index included all the items representing high activation, regardless of their pleasantness. That is, it included all the items in the Activated Pleasant quadrant and the items in the Activated Unpleasant quadrant, as well as the unclassified, ‘border’ items: surprised and no panic. Altogether this index was composed of 51 items.

#### Low activation mood index

This index included all the items representing low activation, regardless of their pleasantness. That is, it included all the items in the Deactivated Unpleasant quadrant and the items in the Deactivated Pleasant quadrant, as well as the unclassified, ‘border’ items: no concern and no surprise. Altogether, this index was composed of 29 items.

### Data collection and analysis

We used the historical quotes database on the Yahoo! Finance website to collect data on all 250 trading days during the year 2000. We chose this year since it contained both up and down markets, generally rising during the early months of the year, becoming more neutral during the middle months, and generally declining during the later months of the calendar year. Therefore, with the year 2000 we could collect consecutive data for one year's time and still capture enough variation to test our hypotheses. However, it should be recognized that the full spectrum of market prices (i.e., the pattern of increases and decreases) was primarily limited to NASDAQ trading during the year. For example, the average overnight move of the NASDAQ (from one day's closing to the next day's opening) was 1%, whereas the average overnight move of the DOW was only .06% and the average overnight move in the S&P was 0.23%. Thus, in order to have sufficient variance to be explained in the overnight movement of stock prices, we limited our hypothesis testing to NASDAQ stock prices.

Our data source consisted of all articles describing market behavior and investor emotions on a particular trading day, which appeared in any U.S. newspaper that is listed in the Lexis-Nexis Academic database (where newspapers used syndicated reports that appeared in several newspapers, we analyzed only one such report. Also, we searched the Wall Street Journal manually, as it does not appear in full in Lexis-Nexis Academic database.) To identify relevant articles, we searched the keywords: stock market *or* Wall Street *and* stocks. We further limited the search by excluding inappropriate articles and materials, such as “Information Bank” abstracts, and company news, as well as non-US market related articles. We then searched the retrieved articles using the emotion keywords presented in Figure 1, including all common derivations of these terms (e.g., fear, feared, fearful, fearing). Four independent raters collected the data. Two raters coded each trading day. Coding was based on a coding scheme emphasizing the particular context in which the emotion terms were mentioned. For example, in our coding scheme, the emotion had to be mentioned regarding the specific trading date (rather than describing a broader time frame, such as a trading week); the emotion term must have been ascribed to a particular party (e.g., traders or financial commentators) experiencing the emotion rather than a general usage, such as “the Great Depression”; the emotion term had to be mentioned in relation to trading, rather than in a different context (such as future predictions unrelated to the specific trading date); and, the emotion term had to have been related to the market as a whole rather than to a specific stock, etc. Full details of the coding scheme appear in Appendix S1. We assessed inter-rater agreement using intra-class correlations (ICC). The ICC(C,2) ranged from 0.44 to 0.96, with an average of 0.78. The raters met to discuss disagreements in coding. If the two raters could not reach a consensus, all four raters would discuss the particular trading day to reach consensus.

We conducted time series analyses to analyze the relationship between mood and overnight changes in stock prices [104], [105]. Specifically, we employed Auto Regressive, Integrated, Moving Average models (ARIMA, [104]), since they can account for trends, seasonality, and autocorrelation that may exist in the data. These models are often used with stock market data. In conducting ARIMA analyses we follow the recommendations by Cromwell, Labys and Terraza [106], Cromwell, Hannan, Labys and Terraza [107], Fuller, Stanton, Fisher, Spitzmuller, Russell, and Smith [108], and Yaffee and McGee [109]. In our sample there were 250 observations (i.e., trading days), which provided sufficient statistical power for testing our hypotheses [109]. Due to space considerations, we do not elaborate here on the details of the ARIMA analyses. Interested readers can receive these details from the authors.

## Results

### Time series model identification

Before performing the time series analyses, the time series models for the dependent and independent variables were identified. The results of the model identification process are presented in Table 1. In this table, the order of the autoregressive and moving average parameters for each independent and dependent variable are listed. For example, the unpleasantness index required fourth order autoregressive parameters, but no moving average parameters. The high activation index, on the other hand, required second order autoregressive parameters and third order moving average parameters. For the next day opening price and the previous day closing price of the NASDAQ, we removed time trends by differencing the opening and closing prices as well as estimating the parameters for the autoregressive and moving average processes. We used the same procedure for the mood indices, but differencing of these independent variables was not needed.

**Table 1:** Order of autoregressive and moving average parameters.

*Variable*	*P*	*Q*
NASDAQ Closing Price	2	2
Deactivated Unpleasant emotion index	5	0
Deactivated Pleasant emotion index	0	3
Activated Unpleasant emotion index	4	0
Activated Pleasant emotion index	1	1
High Activation emotion index	2	3
Low Activation emotion index	1	3
Pleasantness emotion index	3	0
Unpleasantness emotion index	4	0

*Note*. *p* = autoregressive parameter, *q* = moving average parameter.

### Time series analyses for next day opening price

We performed univariate and multivariate transfer function analyses, with the differenced next day opening price as the criterion. The univariate analyses included only one mood index and the prior day closing price as a control variable. We used the univariate analyses to understand the independent relationships that each index showed with the opening price on the next day. The multivariate models included all of the four quadrant mood indices or combinations of the indices as predictors and the prior day closing price as a control variable. The results of the multivariate models were primarily used to test our hypotheses, because these models account for the correlations among the various predictors [110]. However, we also consider the consistency of results between the univariate and multivariate tests in assessing the confirmation or disconfirmation of our hypotheses.

We present the univariate analyses in the first five data columns of Table 2 and the first three data columns in Tables 3 and 4. As seen in the tables, all of the mood indices except the low activation mood index were significantly related to the next day opening price, after controlling for the previous day closing price. Both activated and deactivated pleasant moods were associated with higher next day opening prices (see Table 2). In the same manner, activated and deactivated unpleasant moods were associated with lower next day opening prices (see Table 2). And, as one would expect, univariate analyses using the aggregated indices of pleasant and unpleasant mood (see Table 3) showed the same significant relationships with next day prices as the univariate analyses based on the four mood quadrants. Thus, the results show that mood valance is predictive of congruency in stock prices.

**Table 2:** Transfer Function Results Predicting NASDAQ Opening Price from Previous Day's Mood.

	Prior Day Closing Price Control		Deactivated Pleasant Mood Univariate		Deactivated Unpleasant Mood Univariate		Activated Unpleasant Mood Univariate		Activated Pleasant Mood Univariate		Full Multivariate Model	
	∧		∧		∧		∧		∧		∧	
	B	T-value	B	T-value	B	T-value	B	T-value	B	T-value	B	T-value
*Market*												
Prior day closing price	−.001	−.61	.0007	.45	−.003	−1.53	−.004	−2.08*	.004	4.00**	−.0003	−.19
*Mood*												
Deactivated pleasant mood			150.61	2.55*							175.61	2.05*
Deactivated unpleasant mood					−183.76	−2.61**					230.84	3.09**
Activated unpleasant mood							−154.37	−6.14**			−258.12	−7.06**
Activated pleasant mood									166.07	3.84**	173.06	3.32**
Variance of dependent variable			13378		13378		13378		13378		13378	
Residual variance			13458		13086		12545		13004		10262	
% of Variance Modeled			0.00%		2.18%		6.22%		2.79%		23.29%	

*Notes*. N = 251 days of NASDAQ price data.

* p<.05.

** p<.01.

All analyses include ARIMA(3,0,3) terms.

**Table 3:** Transfer Function Results Predicting NASDAQ Opening Price from Previous Day's Pleasant and Unpleasant Moods.

	Prior Day Closing Price Control		Pleasant Mood Univariate		Unpleasant Mood Univariate		Full Multivariate Model	
	∧		∧		∧		∧	
	B	T-value	B	T-value	_B_	T-value	B	T-value
*Market*								
Prior day closing price	−.001	−.61	−.002	−2.14*	.0008	4.47**	−.0003	−.20
*Mood*								
Pleasant mood			196.46	3.72**			345.49	6.45**
Unpleasant mood					−194.77	−6.25**	−235.72	−6.65**
Variance of dependent variable			13378		13378		13378	
Residual variance			12730		12634		11018	
% of Variance Modeled			4.84%		5.56%		17.64%	

*Notes*. N = 251 days of NASDAQ price data.

* p<.05.

** p<.01.

All analyses include ARIMA(3,0,3) terms.

**Table 4:** Transfer Function Results Predicting NASDAQ Opening Price from Previous Day's High and Low Activation Moods.

	Prior Day Closing Price Control		High Activation Mood Univariate		Low Activation Mood Univariate		Full Multivariate Model	
	∧		∧		∧		∧	
	B	T-value	B	T-value	B	T-value	B	T-value
*Market*								
Prior day closing price	−.001	−.61	0.003	2.45**	−.00004	−.03	0.001	.69
*Mood*								
High activation mood			−153.99	−4.69**			−278.51	−5.45**
Low activation mood					−126.44	−1.26	−490.51	−3.58**
Variance of dependent variable			13378		13378		13378	
Residual variance			12758		14092		12323	
% of Variance Modeled			4.63%		0.00%		7.88%	

*Notes*. N = 251 days of NASDAQ price data.

* p<.05.

** p<.01.

All analyses include ARIMA(3,0,3) terms.

The results for activation were somewhat more complex. Both low and high levels of activation showed a positive and negative relationship with stock prices when paired with the pleasant and unpleasant mood indices, respectively (see data columns 2–4 in table 2). However, independently, a low level of activation was not significantly related to next day opening prices (see data column 3 of Table 4). A high level of activation was significantly and negatively related to next day opening prices (see data column 2 of Table 4). Thus, mood activation level is not a systematic predictor of stock prices.

The closing price on the previous day was not a statistically significant predictor of the next day opening price, independently or in the presence of the collective mood indices (see Table 2). This finding is to be expected given that we removed from the data the autoregressive and moving average processes. The percent of variance explained by each variable is also included in the table. Please note that, for the deactivated pleasant mood index (Table 2) and the low activation index (Table 4), we set the variance to zero because the variance was negative. It is akin to the situation in meta-analysis where one explains more than 100% of the variance (i.e. less than zero residual variance) after accounting for sampling error variance [111].

The last column of Table 2 also shows the results for the multivariate analysis in which we used all mood quadrants to predict next day opening prices. Each of the reported mood indices was a significant predictor of NASDAQ prices. The direction of three of these four relationships was supportive of Hypothesis 1a, that of trend continuance. However, there was a reversal in the effect for deactivated unpleasant mood, changing from a negative relationship in the univariate analysis to a positive relationship in the multivariate analysis. The direction of this quadrant's relationship with opening prices provides some support for trend reversal (Hypothesis 1b), based on the multivariate results. But, given the inconsistency between the univariate and multivariate analyses for this quadrant, we must characterize support for trend reversal as somewhat mixed.

We also include the percent of variance explained by each variable in the first five data columns of Table 2. Time series analyses do not provide traditional indicators of the percent of variance explained (e.g., R^2^). Consistent with Fuller et al. (2003) we computed the variance explained in each model as the sample variance in the dependent variable, minus the residual variance divided by the sample variance. Of all the mood indices, the activated unpleasant mood index explained the greatest amount of variance in NASDAQ opening prices (over 6%). Altogether, the four mood quadrants combined to explain over 23% of the variance in next day opening prices (see bottom row in the last column in Table 2). For the deactivated pleasant mood index, we set the variance to zero because the variance was negative. As was the case for the univariate analyses, closing prices (from the previous day) were not a statistically significant predictor of next day opening prices, independently or in the presence of the various mood indices.

To better understand the relationships between reported mood and market prices and to more closely examine Hypotheses 1 and 2, we conducted two additional transfer function analyses in which we separated the effects of pleasantness and activation level. In the first analysis (see Table 3), we examined the effects of pleasantness and unpleasantness on the next day's opening price, controlling for the previous day's closing price. Both valence indices were statistically significant predictors of market behavior and explained more than 17% of the variance in NASDAQ opening prices. In accordance with Hypothesis 1a, mood valence predicted trend continuance. Specifically, pleasantness demonstrated a positive predictive relationship, such that pleasant mood on day one was associated with higher opening prices on the following trading day. Similarly, unpleasantness demonstrated a negative predictive relationship, such that unpleasant mood on day one was associated with lower opening prices on the following trading day.

In the second transfer function analysis (see Table 4), we examined the effects of high and low activation levels on the next day's opening price, controlling for the previous day's closing price. When both activation indices were simultaneously entered into the analysis, they were both statistically significant predictors of opening NASDAQ prices, hence providing some support for the effect of mood activation levels on market performance. However, support for hypotheses 2a or 2b was rather mixed in the multivariate analysis, since there were negative effects of both high and low activation on stock prices. From the univariate analyses (data columns 2–3 of Table 4), only high activation was a significant predictor of the opening price on the next trading day.

Finally, we considered the role of activation in either trend continuance or reversal. Examining results from the univariate analyses (see the first five columns of Table 2), one can see that activated moods were somewhat stronger predictors of market behavior than were deactivated moods. In fact, the most significant predictors of NASDAQ opening prices were activated unpleasant and activated pleasant moods, both in the direction of trend continuance. The pattern of these data lends some support to the role of activation in amplifying the effects of mood on subsequent stock prices.

## Discussion

Our results showed that mood indices were statistically significant predictors of NASDAQ's opening prices, after controlling for previous day closing prices and time series factors. The specific direction of these relationships was generally in line with Hypothesis 1a. Indices of reported pleasant mood predicted higher opening stock prices, while indices of reported unpleasant mood predicted lower opening stock prices. Examination of the influence of specific mood quadrants showed that both activated and deactivated pleasant moods predicted higher stock prices, while only activated unpleasant mood was a significant predictor of market declines.

Both high and low activation generally predicted lower stock prices, though the strongest relationship appeared to be that of activated unpleasant mood leading to price declines. The driving force of this relationship would appear to be the strong effect of activated unpleasant mood upon stock prices (see univariate and multivariate results in Table 2). Thus, there was at least some support for the notion that activation amplifies the effect of particular mood states.

Because our design and data do not allow us to examine the specific mechanisms behind the relationships between traders' collective mood and market performance, we can only assume that various cognitive processes underlie the effects. For example, reported pleasant moods, such as enthusiasm, hope, joy, calmness, and relief, may influence perception and decision-making processes, as they provide traders the information that the market is safe (e.g., [54], [55]); that market-related events are positive [56]; and that the market poses relatively low risk [70], [71]. Pleasant states can also increase traders' willingness to take higher risks (e.g., [43]), perhaps in the hope of avoiding future regret for not taking advantage of a rising market [112]. These cognitive processes can lead to behavioral reactions, such as a greater willingness of traders to buy into the market so as to maintain their pleasant mood [65], [66].

By the same token, we can also posit that reports of activated unpleasant mood states (such as panic, rage, confusion, and pain) may predict market declines due to similar cognitive mechanisms. Unpleasant collective mood may lead traders to perceive the market and market-related events as negative [56]; that the market is dangerous (e.g., [54], [55]) and lacks opportunity [70]–[72]. Such perceptions may deter traders from taking risks (e.g., [43]), and prompt traders to leave the market [72] in order to repair their unpleasant mood [65], [66] or to avoid future regret for not leaving the market before a further decline ensues [112].

Reports of deactivated unpleasant moods displayed a different pattern of behavior, at least judging by the multivariate results. States such as despair, depression, pessimism, and disappointment were associated with market increases. Because unpleasant mood in and of itself predicted market declines (Table 3), and activation level also predicted market declines (Table 4), it seems that there may be a unique quality to the interaction between unpleasantness and low levels of activation that could precede market increases. Deactivated unpleasant mood can lead traders to manage their moods by taking on greater risk, perhaps in the hope of making gains in the market and improving their mood states (e.g., [94], [95]). Such mood incongruent processes (e.g., [78], [79]) might therefore have led to an increase in stock purchases and contributed to higher stock prices (e.g., [94], [95]).

A growing body of research has shown that not all unpleasant emotions are created equal or have equal consequences [113]. Of particular interest are studies differentiating between cognitive processes involved in similarly toned emotions (e.g., [114], [115]). For example, emotions characterized by appraisal of uncertainty, such as fear, worry, sadness, surprise, and hope, lead to more effortful and systematic cognitive processes, as compared to emotions characterized by appraisals of certainty (e.g., anger, disgust, happiness, and contentment), which generally lead to increased use of stereotypes and heuristics [116]. Similarly, sad people have been found to prefer high-risk/high-reward gambles as a way to repair their moods, whereas anxious people have been found to prefer low-risk/low-reward options so as to reduce uncertainty [113]. Thus, it would be interesting for researchers to examine the level of certainty contained in the deactivated unpleasant mood index, as compared to that in the activated unpleasant index. If there are more uncertainty-related emotions in the unpleasant deactivated index, such a difference might explain the pattern of our results. Another possible explanation for the increase in stock prices following deactivated unpleasant moods could be a more direct influence on stock purchase behavior. For example, it was found that sad people spend more money on purchases as a means of self-enhancement [117]. Because sadness belongs to the deactivated unpleasantness group of emotions [118], it could be that such emotions directly lead to greater spending on stocks, increasing next day stock prices.

### Activation level and market performance

We found that both high and low reported activation levels preceded declines in stock prices. In a somewhat similar vein, Tetlock [39] found that very high or very low levels of a Pessimism Factor predicted high trading volume in the Dow. Rubaltelli, Pasini, Rumiati, Olsen, and Slovic [119] likewise found that extreme levels of pleasant and unpleasant moods (but not moderate mood levels) led to the selling of losing funds. Although we do not know the mood activation levels in these prior studies, it might be the case that such activation played a role in the reported results. This is another interesting question for future research.

Our results are also in line with theoretical models and previous findings showing that high activation can impair decision-making (e.g., [45], [94], [120], [121]), and elicit high risk perception [122]. Although some of these effects were found in pleasant affective states [88], [123], most of these influences of arousal were found particularly strong in unpleasant affective states, thus providing at least some support for the notion that activation amplifies the effect of particular mood states [124], [125]

One way to explain these seemingly counterintuitive findings is that the relationship between activation level and market performance may be curvilinear, such that at extreme levels of activation the market is more likely to decline [120]. Kaufman relied on the Yerkes-Dodson law of optimal arousal. Applying this explanation to our results, we can say that if the activation level of an emotion is far from the optimal level, decision-making will be impaired. Extremely high activation emotions (e.g., panic, exuberance) may require too much cognitive resource to deal with, at the expense of sound decision-making [88], [94]. Extremely low activation emotions (e.g., depression, relaxation) might lead to one's inability or unwillingness to exert vigilant decision-making practices. For now, however, our results do offer some support for Seo et al.'s [126] suggestion that activation can provide as much information to decision makers as valence. For example, Storbeck and Clore [125] suggested that thigh arousal conveys information about the urgency and importance of events.

### The press and other collective mood venues

Whereas our results were based on mood data collected from the traditional written press, new forms of communication seem to be another way by which collective moods can be formed and influence the market. For example, Bollen, Mao, and Zeng [127] investigated how Twitter moods predict the level of the Dow in subsequent days. They found that Twitter data tapping dimensions of happiness and calmness improved predictions of the Dow's movement three days later. However, other mood indices (e.g., a positive-negative sentiment indicator named OpinionFinder, and measures of being alert and kind) had no added value for predicting movement in the Dow. In another Twitter-based study, Zhang, Fuehres, and Gloor [128] found that both pleasant (hope) and unpleasant (fear, worry, anxious, and negative) Twitter moods predicted market declines the next day, and that hope, fear, and worry tweets predicted the market three days after they were posted. In a similar line of research, Gilbert and Karahalios [129] constructed an anxiety index from blog posts dedicated to peoples' reports on their daily lives. Their anxiety index was composed of posts tagged with the words anxiety, worry, and fear. Their index predicted declines in the S&P.

One limitation of these social media studies would seem to be their reliance on non-theoretically based mood indices, without explaining how or why some mood terms predict market activity while other (logically parallel) terms do not. By comparison, in the current study we were much more conservative in terms of mood conceptualization and measurement, relying on a well-accepted circumplex model of emotion. A second limitation of the social media studies may be the relevance of their mood data to the stock market or stock traders. In the current study, we limited the data sample to newspaper reports on the reactions of traders or to descriptions of stock market activity. In contrast, the social media studies have coded for any expression of emotion in communications among individuals, regardless of whether the reference is to business or personal life, and regardless of the age or financial circumstances of the communicator and his/her likelihood of trading stocks. Thus, the relationships between mood and market identified in these studies might be unrelated to the mood of traders, and hence lacks predictive validity. Moreover, our study, using traditional newspaper reports, provides a very conservative test of our hypotheses. We assume that the influence of social media can be much wider than that of newspapers. The fact we were able to support our hypotheses using these data, attests to the capability of our model.

That said, future studies should continue to examine both traditional and social media as well as alternative venues for trading (e.g., on-line trading, day trading, independent trading, or trading via brokers). To the extent that new media increases the flow of communication in quantity and speed, there may be heightened effects for both mood contagion and its influence on financial activity [130].

### Considerations of the current study

One of the advantages of the present investigation is the fact that our mood indices were not likely contaminated by market fundamentals, as often can be the case with measures of investor sentiment [19]. Because we hold constant market prices from the end of the trading day, it can be argued that most important economic news has already been incorporated into closing market prices and therefore controlled. This strengthens our claim that the reported mood of traders does predict stock behavior.

Because the results were largely in line with our trend continuation hypotheses, one might conclude that we simply validated the precepts of momentum (or positive feedback) investment strategies, at least in the very short run (e.g., [131], [132]). Yet, the effects shown in this research are really above and beyond any price momentum, given that prior price movements were statistically controlled. Thus, it is more logical to conclude that the experience and/or observation of moods influences subsequent trading behavior.

A possible critique of the present study is that our data do not directly tap into the collective mood of traders, but consist of journalists' observations and/or interpretations of those moods. While this is certainly a fair criticism, it should also be noted that it is quite likely that traders and individuals associated with the stock market read newspaper articles about the market and/or interact with others who have read these articles. Therefore, regardless of whether “accurate” emotions have been reported by financial journalists, it can be argued that these articles can influence the *perception* of prior market emotion. For example, if the morning paper reported that the stock market and/or traders had shown “fear” and “panic,” these emotions may spread to other investors vicariously. And, even those who are unaffected emotionally may take heed of these emotional states, factoring them into their subsequent investment decisions.

## Conclusion

The findings from this study have both theoretical and practical implications. Probably the most practical or applied implication of our findings concerns the ability of stock traders to make use of these results in their own trading decisions. By reading reports of the collective mood of investors on a given trading day, traders might be able to better predict the direction of the market for the following trading day. However, given the time it took us to code the relevant data (actually reading each article for its appropriate coding of emotion), such procedures would likely be impractical for placing bets on next day trading. To profit from our findings it may therefore be necessary to monitor the mood of the market more quickly, perhaps through a more limited sample of large circulation newspapers. Internet versions of newspapers and financial websites such as Yahoo! Finance and Marketwatch.com could also provide more timely data than traditional newspapers. Yet, it remains an open question whether the effects of emotion will be strong enough to compensate for stock trading expenses. Thus, one possibility for strengthening the effects of emotion would be to concentrate on predicting the prices of speculative stocks (e.g., NASDAQ traded stocks) rather than the market as a whole. Not only would the price variance be larger with more speculative stocks, but it is possible that emotion plays a larger role in the pricing of speculative stocks than with more stable (or value oriented) stocks. These and related conjectures await future research by academics as well as financial experiments by participants in the stock market.

Theoretically, our findings regarding the importance of activation level may be of particular interest, since activation is much less studied than is valence. Researchers from other disciplines can also find our findings illuminating. For example, researchers of economics and behavioral economics can also gain from these findings as they relate to important economic phenomena such as the aggregation of individual decisions in the marketplace. Researchers from organizational psychology and organizational behavior can use these findings as a basis for further examination of the effects of employees' collective mood on behavior in the organizational context, perhaps leading to a broader understanding of factors leading to both organizational and unit performance.

Of interest to researchers of emotions and social psychology are the relationships between collective mood and collective behavior. Because collective behavior is composed of the decision making of individuals, one might look at our findings as spanning multiple levels of analysis. The fact that reported mood of traders predict trading behavior, further underscores the importance of affect in behavior, in this case, at the collective level.

## Supporting Information

Appendix S1 **Coding decision rules.**
(DOCX)
