# Supplementary material for: Mood and the Market: Can Press Reports of Investors' Mood Predict Stock Prices?
Source: PLoS One. 2013 Aug 28;8(8):e72031. doi: 10.1371/journal.pone.0072031 (PMC3756040; doi:10.1371/journal.pone.0072031)
Supplement: Appendix S1 — Coding decision rules. (DOCX) [file pone.0072031.s001.docx]

Appendix

Decision rules for including an emotion word in the data

1. The emotion word should **represent** the mood/emotion of the **stock market** or the **investors** or of **commentators**, **traders**, **analysts** or **other members of the investing community** **on a particular trading date**.
2. If the trading date is clearly embedded in a week or a month discussed in the article, and follows the same pattern of trading as the week or the month in which it is included, we include it.
3. The emotions or states should be a result or a cause or closely associated with the relevant trading activity.
4. The emotions can also refer to the **recent past** (e.g., events that led to the behavior in that trading date, such as “recent events in Russia created panic among traders), **current time**, or **near future** (predictions made by the above commentators, analysts and policy makers).
5. The articles should not refer to older events, unless their relationship to the current state of the market is apparent. A past event is included only if it clearly affects trading on the relevant day.
6. The emotion term can also represent the forecast or the general expectations for the economy by a specific analyst (I am optimistic that we will achieve our goals…)
7. The article should not deal with a specific stock/company or sector (e.g., Tech, pharmaceuticals). One exception to that:
   1. If it is a specific company/sector that triggered an overall market reaction it should be included (e.g. negative earning forecast of IBM cause a panic sale in the market)
8. **“Depression”** and other emotion words should be used **only as describing an emotion**, and not an event (the big depression), verb (prices were depressed), or adjective (prices are depressed).
9. References to the “consumer confidence index” should not count.
10. The topic of the articles should be the stock market. If the article deals with additional things (e.g., housing prices) make sure the emotion word actually refers to the market activity.
11. Make sure the newspaper is American.
12. Make sure an article does not repeat itself in numerous newspapers.
13. Enter number of times the word appears at different articles. If the word repeats itself several times in the same article count it is a single appearance of the word in that article.
14. Be aware of negative mention of the word (e.g., if we are looking for ‘panic,’ make sure the sentence does not talk about ‘no panic.’
15. Descriptions of photos should be included if relevant. We treat them like text.
16. Emotions of one investor/commentator/analyst should be included if they seem representative of the market.
